# Biallelic *C1QBP* Mutations Cause Severe Neonatal-, Childhood-, or Later-Onset Cardiomyopathy Associated with Combined Respiratory-Chain Deficiencies

**DOI:** 10.1016/j.ajhg.2017.08.015

**Published:** 2017-09-21

**Authors:** René G. Feichtinger, Monika Oláhová, Yoshihito Kishita, Caterina Garone, Laura S. Kremer, Mikako Yagi, Takeshi Uchiumi, Alexis A. Jourdain, Kyle Thompson, Aaron R. D’Souza, Robert Kopajtich, Charlotte L. Alston, Johannes Koch, Wolfgang Sperl, Elisa Mastantuono, Tim M. Strom, Saskia B. Wortmann, Thomas Meitinger, Germaine Pierre, Patrick F. Chinnery, Zofia M. Chrzanowska-Lightowlers, Robert N. Lightowlers, Salvatore DiMauro, Sarah E. Calvo, Vamsi K. Mootha, Maurizio Moggio, Monica Sciacco, Giacomo P. Comi, Dario Ronchi, Kei Murayama, Akira Ohtake, Pedro Rebelo-Guiomar, Masakazu Kohda, Dongchon Kang, Johannes A. Mayr, Robert W. Taylor, Yasushi Okazaki, Michal Minczuk, Holger Prokisch

**Affiliations:** 1 Department of Pediatrics, University Hospital Salzburg, Paracelsus Medical University, 5020 Salzburg, Austria; 2 Wellcome Centre for Mitochondrial Research, Institute of Neuroscience and Institute for Cell and Molecular Biosciences, Newcastle University, Newcastle upon Tyne NE2 4HH, UK; 3 Research Center for Genomic Medicine, Saitama Medical University, Saitama 350-1241, Japan; 4 Diagnostics and Therapeutics of Intractable Diseases, Intractable Disease Research Center, Juntendo University, Graduate School of Medicine, Tokyo 113-8421, Japan; 5 Medical Research Council Mitochondrial Biology Unit, University of Cambridge, Wellcome Trust, MRC Building, Cambridge CB2 0XY, UK; 6 Institute of Human Genetics, Technische Universität München, 81675 Munich, Germany; 7 Institute of Human Genetics, Helmholtz Zentrum München, 85764 Neuherberg, Germany; 8 Department of Clinical Chemistry and Laboratory Medicine, Graduate School of Medical Sciences, Kyushu University, 3-1-1 Maidashi, Higashi-ku, Fukuoka 812-8582, Japan; 9 Howard Hughes Medical Institute, Department of Molecular Biology, Center for Genome Medicine, Massachusetts General Hospital, Boston, MA 02114, USA; 10 Broad Institute of MIT and Harvard, Cambridge, MA 02142, USA; 11 DZHK (German Centre for Cardiovascular Research), partner site Munich Heart Alliance, 80802 Munich, Germany; 12 South West Regional Metabolic Department, Bristol Royal Hospital for Children, Bristol BS1 3NU, UK; 13 Department of Neurology, Columbia University Medical Center, New York, NY 10032-3784, USA; 14 Neuromuscular Unit, Fondazione IRCCS Ca’ Granda, Ospedale Maggiore Policlinico, 20122 Milan, Italy; 15 Neuroscience Section, Department of Pathophysiology and Transplantation, Dino Ferrari Center, University of Milan, IRCCS Foundation Ca’ Granda, Ospedale Maggiore Policlinico, 20122 Milan, Italy; 16 Department of Metabolism, Chiba Children’s Hospital, Chiba 266-0007, Japan; 17 Department of Pediatrics, Faculty of Medicine, Saitama Medical University, Saitama 350-0495, Japan; 18 Graduate Program in Areas of Basic and Applied Biology, University of Porto, 4099-002 Porto, Portugal

**Keywords:** mitochondria, multiple mtDNA deletions, oxidative phosphorylation, lactate, progressive external ophthalmoplegia, PEO, myopathy, MAM33, p32

## Abstract

Complement component 1 Q subcomponent-binding protein (C1QBP; also known as p32) is a multi-compartmental protein whose precise function remains unknown. It is an evolutionary conserved multifunctional protein localized primarily in the mitochondrial matrix and has roles in inflammation and infection processes, mitochondrial ribosome biogenesis, and regulation of apoptosis and nuclear transcription. It has an N-terminal mitochondrial targeting peptide that is proteolytically processed after import into the mitochondrial matrix, where it forms a homotrimeric complex organized in a doughnut-shaped structure. Although C1QBP has been reported to exert pleiotropic effects on many cellular processes, we report here four individuals from unrelated families where biallelic mutations in *C1QBP* cause a defect in mitochondrial energy metabolism. Infants presented with cardiomyopathy accompanied by multisystemic involvement (liver, kidney, and brain), and children and adults presented with myopathy and progressive external ophthalmoplegia. Multiple mitochondrial respiratory-chain defects, associated with the accumulation of multiple deletions of mitochondrial DNA in the later-onset myopathic cases, were identified in all affected individuals. Steady-state C1QBP levels were decreased in all individuals’ samples, leading to combined respiratory-chain enzyme deficiency of complexes I, III, and IV. *C1qbp*^*−/−*^ mouse embryonic fibroblasts (MEFs) resembled the human disease phenotype by showing multiple defects in oxidative phosphorylation (OXPHOS). Complementation with wild-type, but not mutagenized, *C1qbp* restored OXPHOS protein levels and mitochondrial enzyme activities in *C1qbp*^*−/−*^ MEFs. C1QBP deficiency represents an important mitochondrial disorder associated with a clinical spectrum ranging from infantile lactic acidosis to childhood (cardio)myopathy and late-onset progressive external ophthalmoplegia.

## Introduction

Mitochondrial disorders are an extremely heterogeneous group of inborn errors of metabolism and encompass a wide range of clinical presentations, such that approximately 300 disease-associated genes have been identified to date.^1^, ^2^ Mitochondrial dysfunction mainly affects organs with high energy requirements, such as the brain, central nervous system, muscle, and heart. The broad clinical and genetic presentation of mitochondrial disorders makes the molecular diagnosis challenging. Mutations can directly affect oxidative phosphorylation (OXPHOS) subunits or indirectly impair OXPHOS activity by disturbing mitochondrial homeostasis. Next-generation sequencing techniques (gene panels and exome and genome sequencing) are proving to be an appropriate tool for the diagnosis of this broad clinical group. However, any diagnostic approach continues to rely upon deep clinical phenotyping in association with the evaluation of OXPHOS enzymes in tissues of affected individuals.^3^, ^4^, ^5^, ^6^ Combined defects of complexes I, III, IV, and V are typically due to deficiencies involving the homeostasis of mitochondrial DNA (mtDNA), including defects in replication, RNA metabolism, and translation.^1^, ^7^ Moreover, mtDNA rearrangements can lead to combined OXPHOS deficiencies; single, large-scale mtDNA deletions, predominantly found in sporadic cases, are associated with Pearson syndrome (MIM: 557000), Kearns-Sayre syndrome (MIM: 530000), or progressive external ophthalmoplegia (PEO; OMIM phenotypic series PS157640), as well as late-onset PEO due to Mendelian-driven multiple mtDNA deletions, which have been observed in >20 genetically distinct disorders^8^ (also see GeneReviews in Web Resources). In addition, cofactor deficiencies and further defects of mitochondrial homeostasis—including mitochondrial biogenesis, lipid metabolism, protein import, fission and fusion, and quality control—can result in a deficiency of more than one OXPHOS enzyme.^2^

One protein involved in mitochondrial homeostasis is complement component 1 Q subcomponent-binding protein (C1QBP; also known as p32). It is an evolutionary conserved and ubiquitously expressed multifunctional protein and has been reported to be a predominantly mitochondrial matrix protein involved in inflammation and infection processes, mitochondrial ribosome biogenesis, regulation of apoptosis and nuclear transcription, and pre-mRNA splicing.^9^, ^10^, ^11^, ^12^, ^13^, ^14^, ^15^ By analyzing a C1QBP-knockout (KO) mouse model, we have previously demonstrated that a main function of C1QBP is a combined respiratory-chain complex deficiency due to severely impaired mitochondrial protein synthesis.^16^ Furthermore, the *Saccharomyces cerevisiae* ortholog of human C1QBP, MAM33 (mitochondrial acidic matrix protein 33), has been shown to localize to the mitochondrial matrix.^17^ MAM33-deficient yeast cells show a disturbed maintenance of the mitochondrial genome, impairment of mitochondrial ATP synthesis, and growth deficiency.^17^, ^18^ The latter can be restored by the introduction of human *C1QBP* cDNA, which demonstrates the evolutionarily conserved function of C1QBP homologs among eukaryotes.^18^ In line with the complementation in yeast and findings in mice, human C1QBP-knockdown (KD) cells also exhibit reduced synthesis of mtDNA-encoded OXPHOS polypeptides.^19^

Here, we report four individuals from unrelated families affected by biallelic mutations in *C1QBP* (MIM: 601269). They present with multiple OXPHOS deficiencies and a clinical spectrum ranging from infantile lactic acidosis, childhood- or adulthood-onset (cardio)myopathy, and PEO.

## Subjects and Methods

All studies were completed according to local ethical approval of the institutional review boards of Technische Universität München, the University of Milan, the National Research Ethics Service Committee North East – Newcastle & North Tyneside 1, and Saitama Medical University. In agreement with the Declaration of Helsinki, all individuals or their guardians gave written informed consent before undergoing evaluation and testing, which was approved by the ethical committees of the centers participating in this study, where biological samples were obtained.

### Subjects

Individual 1 (S1; family 1 individual II-2) was a boy who died at day 18 after experiencing asymmetric ventricular cardiomyopathy, congenital nephrosis, hypothyroidism, and encephalopathy with multiple hemorrhagic events (Table 1). He was born by spontaneous vaginal delivery to healthy, unrelated British parents after in vitro fertilization at 34 weeks + 2 days of gestation. His twin brother is unaffected. Oligohydramnios was referenced in the antenatal history, and a swollen face, hands, and feet were noticed at birth. On the third day, he presented with prolonged cardiorespiratory arrest. He was resuscitated for 2 hr by both mechanical (chest compression) and pharmacological (adrenaline, sodium bicarbonate, atropine, and calcium chloride) treatments and was intubated and ventilated. After resuscitation, he was admitted to pediatric intensive care unit with severe metabolic acidosis (plasma lactate: 21 mmol/L, normal: 0.5–2.5 mmol/L; base excess [BE]: −18.4 mmol/L, normal: −2 to +2 mmol/L), initial signs of kidney failure (anuria; albumin: 15 g/L, normal: 35–40 g/L; urea: 6.1 mmol/L, normal: 0.8–5.5 mmol/L), and a poor general condition (unconscious, not responsive to stimuli, and fixed and dilated pupils). An increased level of troponin suggested severe myocardial damage secondary to his arrest event, and echocardiography revealed a dilated and poorly functioning left ventricle. He required additional cardiovascular and metabolic support and a blood transfusion. Neurological investigations demonstrated extensive brain damage: electrical discharges defined as suppression bursts and subclinical seizures were recorded by electroencephalography, and brain MRI showed global cerebral edema with a loss of differentiation between gray and white matter, a loss of definition of the basal ganglia on T2-weighted images, multiple areas of hemorrhage in the bilateral subdural region over both cerebral convexities and in the posterior fossa, and subarachnoid hemorrhage in the Sylvian fissures and lateral ventricles. Although he presented with signs and symptoms of congenital nephrosis, his kidney ultrasound showed only general hyperechogenicity. During the clinical course, his neurological conditions slightly improved: he was able to open his eyes and respond to stimuli, and he showed some movement of the legs and arms in the following days. However, lactic acidosis persisted, anuria was resistant to pharmacological treatment, and he also developed hyperkalemia and hyperphosphatemia after blood transfusion, which required peritoneal dialysis (day 4). The clinical course was also complicated by thrombocytopenia on day 6 (which required platelet transfusion), evidence of disseminated intravascular coagulopathy on day 7 (which was treated with fresh frozen plasma and cryoprecipitate), and peritonitis on day 9 (which was treated with antibiotics). The cause of death on day 18 was respiratory insufficiency. Kidney histology on autopsy tissue showed multifocal and diffuse cortical necrosis, multifocal pyramid necrosis, confluent recent hemorrhages surrounding the pyramids, scattered cortical tubular microcysts, and partially or completely sclerosed glomeruli. The number of glomeruli was slightly higher than in normal kidney tissue and showed a different degree of mesangial proliferation. There were also some fibrin thrombi in the glomerular capillaries. Necrosis and multifocal areas of hemorrhage were also present in the lungs, adrenals, spleen, and testes. Histological examinations of heart autopsy tissue revealed the presence of a small recent infarct of the anterior papillary muscle and some hemorrhages and sparse neutrophils around a small recent fibrous scar of the papillar muscle. The thymus showed marked atrophy. The cortex, medulla, and crowded Hassal’s corpuscles were not distinguishable. A pronounced lymphocyte depletion was present.

**Table 1:** Genetic and Clinical Findings in Individuals with *C1QBP* Mutations

	**Proband**			
	**S1**	**S2**	**S3**	**S4**
*C1QBP* variant (GenBank: NM_001212.3)	c.[557G>C];[612C>G]	c.[739G>T];[c.824T>C]	c.[823C>T];[823C>T]	c.[562_564delTAT];[562_564delTAT]
C1QBP variant (GenBank: NP_001203.1)	p.[Cys186Ser];[Phe204Leu]	p.[Gly247Trp];[Leu275Pro]	p.[Leu275Phe];[Leu275Phe]	p.[Tyr188del];[Tyr188del]
Origin	European descent	Asian descent	European descent	European descent
Age of onset	4 days	birth	5 years	57 years
Gender	male	female	male	male
Current age or age of death	18 days (deceased)	4 days (deceased)	22 years (alive)	70 years (deceased)
Antenatal findings	oligohydramnios, oedematus feet, face and hands	IUGR, oligohydramnios	–	–
Plasma metabolic test results	lactate: 21 mmol/L (normal: 0.5–2.5)	lactate: 20 mmol/L (normal: 0.5–2.5)	lactate: 3.2 mmol/L (normal: 0.5–2.5); CPK: 566 U/L (normal: 38–174); Met: 56.2 μmol/L (normal: 14.4–36); Tyr: 145 μmol/L (normal: 41.8–108)	normal

**Clinical Signs and Symptoms**				

Heart	cardiorespiratory arrest, asymmetric left ventricular cardiomegaly	cardiomegaly	left ventricular hypertrophy	left ventricular hypertrophy
Liver	–	hepatomegaly	increased transaminases	–
CNS	multiple cortical, ventricular, and subdural hemorrhages and cerebral edema, burst suppression-like electrical discharges, suclinical seizures	–	NA	NA
PNS	NA	NA	sensory peripheral neuropathy	diffuse neurogenic abnormalities and focal myogenic in the gluteus maximus
Kidney	congenital nephrosis	–	–	NA
Muscle	NA	NA	exercise intolerance with fatigue and vomiting	exercise intolerance, weakness
Eye	NA	NA	astigmatism, amblyopia, ptosis, PEO	ptosis, progressive external opthalmoplegia
Other	hypothyroidism, disseminated intravascular coagulopathy	NA	NA	post-traumatic depression, diabetes, sensorineural hearing loss

Abbreviations are as follows: NA, not available; CPK, creatine phosphokinase; Met, methionine; Tyr, tyrosine; IUGR, intrauterine growth restriction; CNS, central nervous system; and PNS, peripheral nervous system.

Individual 2 (S2; family 2 individual II-1) was a girl who died at 4 days of age after suffering from cardiomegaly and lactic acidosis. She was the first child of healthy, non-consanguineous Japanese parents. Pregnancy was complicated by intrauterine growth restriction and oligohydramnios. She was born pre-term (33 weeks of gestation) by emergency cesarean section as a result of fetal heart-rate failure. In the immediate postnatal period, she developed metabolic acidosis (BE: −25 mmol/L, normal: −2 to +2 mmol/L) and an increased level of lactic acid (9.0 mmol/L at day 0, 19.8 mmol/L at day 1, >20 mmol/L at day 3, and 19.6 mmol/L at day 4; normal: 0.5–2.5 mmol/L). She became unconscious and required ventilator support. Cardiomegaly with no ventricular or septic hypertrophy was diagnosed by heart ultrasound and a thoracic X-ray scan. A general examination revealed that she also presented with mild hepatomegaly, and histological examination of a liver biopsy showed marked lipid accumulation. Additional neurological signs and symptoms were not reported, and brain ultrasound did not reveal any abnormalities. She died on the fourth day of life because of cardiorespiratory insufficiency.

Individual 3 (S3; family 3 individual II-2) is a 22-year old man with myopathy, asymmetric ventricular cardiomyopathy, PEO, and ptosis. He was born after an uneventful pregnancy to non-consanguineous Austrian parents. His sister, who is 3 years older, is healthy. Pregnancy and peri- and postnatal adaptations were unremarkable. Birth weight, length, and head circumference were within normal limits. His early milestone acquisitions were appropriate for his age. He presented with bilateral hyperopic astigmatism and amblyopia, a first sign of eye muscular weakness, at 5 years of age and exercise intolerance with fatigue, episodes of vomiting, and elevated levels of creatine phosphokinase (CPK) 1 year later. Left ventricular cardiomyopathy was also diagnosed at 8 years of age after heart ultrasound screening. The clinical phenotype and increased level of plasma lactate (3.2 mmol/L, normal: 0.5–2.2 mmol/L) were suggestive of a mitochondrial disorder. Treatment with L-carnitine, riboflavin, and coenzyme Q was initiated but showed only initial and partial improvement. The clinical course appeared slowly progressive with the development of mild ptosis at 14 years of age and PEO at 19 years of age. Transaminase levels were repeatedly elevated (aspartate transaminase: 73 U/L, normal: 10–50 U/L; alanine transaminase: 61 U/L, normal: 10–50 U/L), and liver ultrasound showed no structural changes. At the last evaluation, a nerve conduction study revealed subclinical signs of sensory peripheral neuropathy, and scotopic and photopic bilateral electroretinography showed low amplitudes. Assessment of exercise intolerance by bicycle ergometry confirmed premature fatigue and an increased level of plasma lactate (basic lactate: 6.1 and 19.1 mmol/L after exercise, normal: 0.5–2.2 mmol/L) and CPK (basic CPK: 386 and 566 U/L after exercise, normal: 38–174 U/L). The cardiac status was stable without progression. He was investigated for hearing and kidney functionality, which were shown to be normal. Although he has generalized weakness, he is not compromised in his daily functionality.

Individual 4 (S4; family 4 individual II-2) was a 61-year-old man with late-onset PEO and myopathy. He was born at term as the second of ten brothers and sisters to healthy, non-consanguineous Italian parents after an uneventful delivery. No family history of neuromuscular disorders or other disorders, except for the premature postnatal death of one sister, has been reported. Early development and milestone acquisition were referenced as normal. At the age of 57 years, he was admitted to a psychiatric hospital because of post-traumatic depression that required intensive pharmacological and psychotherapy intervention. He was also diagnosed with diabetes and started treatment with gliclazide. The onset of neuromuscular weakness was detected at 59 years of age, and rapidly progressive exercise intolerance limited his functionality: he was not able to stand for long periods of time or raise his arms. He was admitted to the hospital at 61 years of age because of the development of ptosis, severe constipation, and weight loss (10 kg in 5 months). At the neurological examination, he presented with important bilateral eyelid ptosis (left > right), PEO in all directions, mild to moderate proximal weakness, mild and diffuse hypotrophy, and an unstable gait due to lower-limb weakness. Electromyography showed diffuse signs of chronic neurogenic rearrangement and focal myogenic signs in the gluteus maximus. A metabolic workup including plasma lactate, creatine kinase, transaminases, and thyroid function was normal. He was extensively investigated, and additional clinical findings were identified: left ventricular hypertrophy, left ventricular overload, and signs of previous myocardial infarction by electrocardiography; bilateral and symmetric sensorineural hypoacusia by audiometry; and multiple gastric erithematous areas by esophagogastroduodenoscopy. Proband S4 and his family refused additional investigations. He died in his 70s for an unknown reason.

### Exome Sequencing

We applied next-generation exome sequencing with whole-exome sequencing (WES) or a mitochondrial exome library (“MitoExome”) to the individuals’ DNA extracted from peripheral blood. Probands S1–S3 were investigated by WES.^4^, ^5^, ^20^ In brief, coding regions were enriched with a SureSelect Human All Exon V5 Kit (Agilent) or TruSeq (Illumina) and then sequenced as 100-bp paired-end runs on an Illumina HiSeq 2000, 2500, or 4000. Reads were aligned to the human reference genome (UCSC Genome Browser build hg19) with the Burrows-Wheeler Aligner (v.0.7.5 a).^21^ Single-nucleotide variants and small insertions and deletions (indels) were detected by SAMtools (v.0.1.19). Proband S4 was investigated by MitoExome as previously described,^22^ and only rare recessive variants in highly conserved amino acid residues were considered pathogenic by PolyPhen-2 or SIFT (Table S1).

We used Sanger sequencing to confirm the identified mutations and test the carrier status of unaffected family members.

### mDNA Analysis

mtDNA was amplified with two primer pairs, giving a 16,147 bp (F1) or 15,679 bp (F2) fragment. 5 μL of the amplified mtDNA was loaded onto a 0.7% agarose gel and separated at 80 V for 1 hr. The marker λ/HindIII (the bacteriophage λ digested by HindIII) was used.^23^ Southern blot was performed as previously described.^24^ mtDNA copy number was determined by quantitative real-time PCR with four different nuclear and two mitochondrial PCR amplicons.^25^ The relative amount of mtDNA versus nuclear DNA was calculated by the 2^−ΔΔCT^ method.^25^

### OXPHOS Enzyme Activities

Muscle tissues (20–100 mg) were homogenized in extraction buffer (20 mM Tris-HCl [pH 7.6], 250 mM sucrose, 40 mM KCl, and 2 mM EGTA). The post-nuclear supernatant isolated by centrifugation at 600 × *g* and containing the mitochondrial fraction was used for measurement of enzyme activities. OXPHOS enzyme and citrate synthase (CS) activity was measured spectrophotometrically as previously described.^26^, ^27^, ^28^, ^29^

### Western Blot Analysis

Skeletal muscle and fibroblast homogenates were obtained according to previously described methodologies.^30^ 30–40 μg (S1–S3) and 20 μg (S4) of whole-cell protein extracts were separated by SDS polyacrylamide (12%) electrophoresis and then wet transferred to polyvinyl difluoride (PVDF) membranes. For S4, a 4%–12% gradient gel was used. Immunological detection of proteins was carried out with the following primary antibodies: C1QBP (ab24733, Abcam), β-actin (A1978, Sigma), α-tubulin (ab7291, Abcam), and OXPHOS complex-specific antibodies (NDUFS3 [ab14711, Abcam], NDUFB8 [ab110242, Abcam], NDUFA9 [MS111, Molecular Probes], SDHA [459200, MitoSciences], SDHB [ab14714, Abcam], UQCRC2 [ab14745, Abcam], COXI [ab14705, Abcam], COXII [ab110258, Abcam], COXIV [ab14744, Abcam], and ATP5A [ab14748, Abcam]). Species-appropriate horseradish-peroxidase-conjugated secondary antibodies (DAKO, P0399, and P0260) were used.

### BN-PAGE Analysis

For blue-native polyacrylamide-gel electrophoresis (BN-PAGE), n-dodecyl β-d-maltoside-solubilized mitochondrial membranes were prepared from isolated fibroblasts and muscle mitochondria according to previously described methodologies.^30^, ^31^ In brief, 100 μg of mitochondrial extracts was loaded onto a 4%–16% native polyacrylamide BisTris gradient gel (Life Technologies) and separated electrophoretically. For western blot analysis, samples separated by BN-PAGE were transferred onto PVDF membranes and subjected to the following primary antibodies: NDUFB8 (complex I [CI]), SDHA (complex II [CII]), UQCRC2 (complex III [CIII]), COXI (complex IV [CIV]), and ATP5A (complex V [CV]).

### Immunofluorescence Staining

Immunofluorescence was performed according to a modified version of the previously published protocol.^32^ Fibroblasts were grown on coverslips, washed with PBS, and fixed with 4% formaldehyde for 15 min at room temperature. After additional washes with PBS, coverslips were permeabilized with 1% Triton X-100 for 5 min, washed, and blocked in 5% fetal bovine serum (FBS) for 1 hr at room temperature. A solution of primary antibodies (1:500 polyclonal rabbit anti-TOM20, sc-11415, Santa Cruz; 1:250 monoclonal mouse anti-C1QBP, ab24733, Abcam) in 5% FBS was prepared and used for incubating the coverslips for 1 hr at room temperature. After PBS washings, coverslips were incubated with secondary antibodies (1:1,000 Alexa-Fluor-488-conjugated goat anti-mouse IgG, A11001, Invitrogen; 1:1,000 Alexa-Fluor-594-conjugated goat anti-rabbit IgG, A11012, Invitrogen) in 5% FBS for 1 hr at room temperature while being protected from light. After the final washes, coverslips were mounted (ProLong Gold antifade reagent with DAPI, P36941, Invitrogen) on glass slides. Microscopy was carried out with a Zeiss LSM 880 confocal microscope, and acquired images were processed with ImageJ.^33^

### High-Resolution Respirometry

Mouse embryonic fibroblasts (MEFs) were seeded at a density of 20,000 cells/well in 80 μL of culture media in an XF^e^ 24-well cell-culture microplate (Seahorse Bioscience) and incubated overnight at 37°C in 5% CO_2_. Culture medium was replaced with 180 μL of bicarbonate-free DMEM, and cells were incubated at 37°C for 30 min before measurement. The oxygen-consumption rate (OCR) was measured with an XF^e^ 24 Extracellular Flux Analyzer (Seahorse Biosciences) and determined (1) with no additions (basal respiration), (2) after the addition of 0.5 μM oligomycin (indicating ATP production), (3) after the addition of 1 μM carbonyl cyanide 4-(trifluoromethoxy) phenylhydrazone (maximal respiration), and (4) after the addition of 1 μM rotenone and antimycin A (non-mitochondrial respiration) (additives were purchased from Sigma at the highest quality). The difference in OCR after the addition of oligomycin and the non-mitochondrial respiration specify proton leakage, and the difference between basal and maximal respiration determines spare capacity.

### Construction and Validation of C1QBP Expression Vector

The mouse C1QBP variants p.Glu244Trp and p.Leu272Pro (corresponding to the human variants p.Gly247Trp [c.739G>T] and p.Leu275Pro [c.824T>C], respectively, found in probands S2 and S3) were generated from pcDNA3-p32-IRES-GFP by PCR-based site-directed mutagenesis and confirmed by sequencing. To examine protein expression, we transfected *C1qbp*-KO MEFs with the *C1qbp* expression vector by using the transfection reagent Lipofectamine 2000 (Invitrogen) according to the manufacturer’s instructions. GFP expression served as an internal control.

### mRNA Quantification

Total RNA from wild-type (WT) MEFs and *C1qbp*-KO MEFs transfected with various *C1qbp* expression vectors was isolated with an RNeasy Mini Kit (QIAGEN) according to the manufacturer’s instructions. The concentration and purity of total RNA were measured on a NanoDrop spectrophotometer (NanoDrop Technologies). Reverse transcription of 1 μg of total RNA was performed with a PrimeScript RT Reagent Kit (TAKARA). Mouse *C1qbp* mRNA and mouse 18S ribosomal RNA (control) were detected by quantitative PCR with SYBR Premix Ex Taq II (TAKARA) and a thermal cycler (StepOnePlus, Applied Biosystems). Primers were as follows: 5′-CGCGGTTCTATTTTGTTGGT-3′ (18S rRNA forward), 5′-AGTCGGCATCGTTTATGGTC-3′ (18S rRNA reverse), 5′-GGCCTTCGTTGAATTCTTGA-3′ (*C1qbp* mRNA forward), and 5′- GCCTCATCTTCGTGTCCAAT-3′ (*C1qbp* mRNA reverse). An unpaired Student’s t test was used to determine statistical differences between two groups. Values of ^∗^p < 0.05, ^∗∗^p < 0.01, and ^∗∗∗^p < 0.005 were considered to be statistically significant.

## Results

### Exome Sequencing and Variant Prioritization

**Figure 1:**
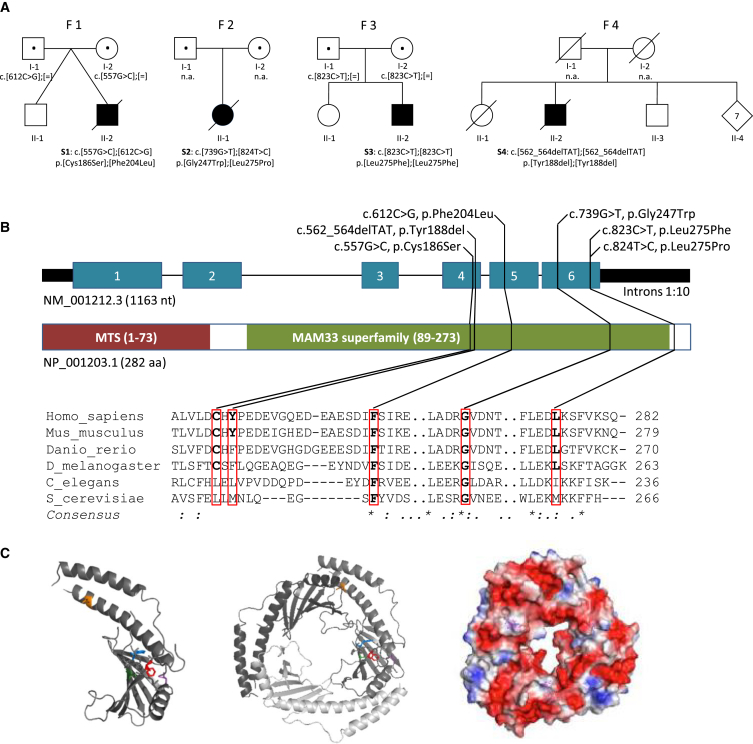
C1QBP Variants and Gene and Protein Structure (A) Pedigrees of the investigated families (S1–S4) affected by recessively inherited C1QBP variants. Affected individuals are indicated by closed symbols. Both variants in proband S2 have been confirmed to be compound heterozygous by phasing of WES data. (B) Gene structure with exons and introns shows the localization of the investigated gene variations. Conservation of the affected amino acid residues is presented in the alignment of homologs across different species. Exons are highlighted in blue. The size of the introns was reduced 10-fold. MTS is the mitochondrial target sequence. MAM33 (mitochondrial acidic matrix protein 33) is the *Saccharomyces cerevisiae* homolog of C1QBP. (C) Inspection of the protein structure was performed with PyMOL (PDB: 1P32). A monomer is presented on the left, and the trimer is in the center. The electrostatic field of the trimer is indicated to the right (negative polarity, red; positive polarity, blue). Affected residues are colored in one of the monomers: Cys186, green; Tyr188, blue; Phe204, red; Gly247, magenta; and Leu275, orange.

Clinical research centers from Austria, Germany, Japan, the UK, and the US independently achieved and subsequently shared results from WES or MitoExome sequencing analysis on individuals with suspected mitochondrial disease. WES of probands S1–S3 failed to identify likely or known disease-associated genetic variants but did reveal rare biallelic variants (minor allele frequency [MAF] < 0.1% in public and in-house databases) in *C1QBP* (GenBank: NM_001212.3). Screening of our in-house database of more than 10,000 WES datasets of individuals with non-mitochondrial disease revealed no additional individual with biallelic rare variants in *C1QBP*. Filtering for genes coding for mitochondrial proteins in probands S1–S3 revealed that *C1QBP* was the only gene with biallelic variants.^34^ Likewise, MitoExome sequencing prioritized biallelic variants in *C1QBP* in proband S4. Altogether, we identified four individuals harboring recessive variants in *C1QBP* (Table 1 and Figure 1A). Although none of the identified variants are predicted to cause a loss of function, all are predicted to be pathogenic (Figure 1B and Table S1). Human C1QBP (UniProt: Q07021) forms a homotrimer arranged into a doughnut-shaped structure with an unusually asymmetric charge distribution on the surface. The variants p.Cys186Ser, p.Gly247Trp, and p.Leu275Pro are all localized in important structural domains of the protein. Specifically, amino acid residue Cys186 is localized on the β5 strand of the protein, Gly247 is located on the hydrogen-bonded turn of the protein, and Pro275 is located on the αC helix (Figure 1C). In contrast, p.Phe204Leu and p.Tyr188del are localized on the coiled-coil region between the β5 and β6 strands.^9^ All *C1QBP* variants have been confirmed by Sanger sequencing. Homozygosity or compound heterozygosity was confirmed by segregation analysis in families 1 and 3 and by cloning and sequencing of genomic DNA in proband S2, whereas no DNA was available from the parents of proband S4 for validation of the deletion’s homozygosity. The *C1QBP* variants found in proband S2 have previously been suggested as likely candidates but have not been validated so far.^4^

### Mitochondrial Respiratory-Chain Activities

The identification of rare, biallelic *C1QBP* variants in four individuals with clinically and biochemically confirmed mitochondrial disease adds further evidence for their functional relevance. Typical of mitochondrial disorders, the *C1QBP* defect resulted in a spectrum of manifestations that ranged from infantile lactic acidosis (probands S1 and S2) to childhood myopathy (proband S3) to late-onset myopathy with PEO (proband S4). However, in all individuals, cardiac symptoms were present, and respiratory-chain activities in muscle or liver homogenates showed a severe combined deficiency of respiratory-chain activities (complexes I, III, and IV). This was accompanied by an increased level of mitochondrial mass index (CS) in the muscle of probands S3 (511 mUnits/mg protein; normal: 150–325 mUnits/mg protein) and S4 (151 mUnits/mg protein; normal: 137 ± 15 mUnits/mg protein; see Table S2).

Enzymatic activities of liver homogenates from proband S2 revealed a more general downregulation of the OXPHOS complexes. In addition to the complex I (to 5%), III (to 14%), and IV (to 11%) deficiency, a reduction in complex II (to 37%) activity was also found (Table S2). In addition, decreased COX histochemical activity was found in proband S1 (affecting up to 75% of all muscle fibers); occasional COX-deficient fibers were observed in muscle from probands S3 and S4, notably at levels above those expected to be detected in age-matched control individuals as a result of somatic mtDNA mutation. Ragged-red fibers were present in the muscle of probands S1 and S3 (Table S2). Histopathological analysis of the muscle of proband S4 showed moderate variability in fiber size, a moderate increase in fiber splitting and central nuclei, and a slight increase in connective tissue.

The identification of four different alleles in four affected individuals with a similar clinical and biochemical phenotype from four families establishes *C1QBP* variants as confidently implicated in recessively inherited mitochondrial disease.

### C1QBP and OXPHOS Protein Levels

**Figure 2:**
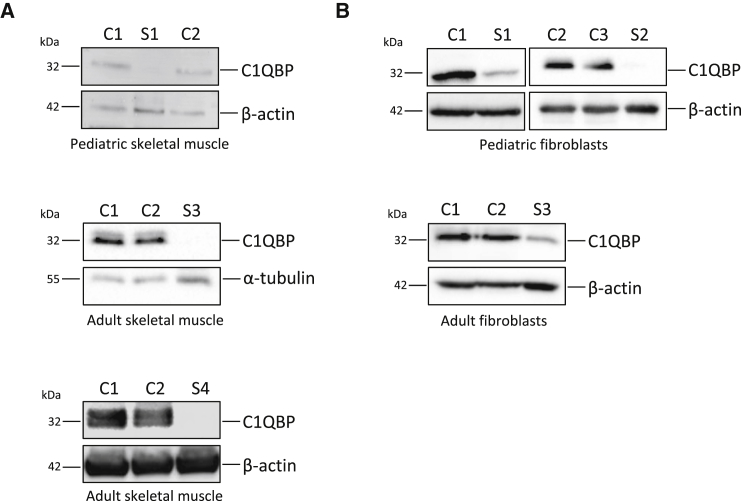
Western Blot Analysis of C1QBP Cell lysates isolated from (A) the skeletal muscle of affected probands S1, S3, and S4 and (B) fibroblasts from probands S1–S3 and age-matched control individuals (C1 and C2) were analyzed. Fibroblasts from proband S4 and skeletal muscle from proband S2 were not available. β-actin and α-tubulin were used as loading controls. All experiments were repeated at least two times, and representative images are shown. Number of repeats: (A) n = 3 (S1) and n = 2 (S3 and S4); (B) n = 2 (S1–S3).

Given that previous studies have reported C1QBP as a mitochondrial-matrix protein, we wanted to validate the localization of C1QBP in mitochondria. Immunocytochemical staining using C1QBP-specific antibodies confirmed the mitochondrial localization of the protein (Figure S1).^9^, ^13^, ^17^ Given that all identified *C1QBP* variants affect only one amino acid, the consequences on protein stability were unclear. We therefore investigated available skeletal-muscle biopsy material and primary fibroblast cultures established from individuals to analyze the significance of *C1QBP* variants in disease. Western blot analysis showed that C1QBP levels were not detectable in muscle (Figure 2A) and considerably decreased in fibroblasts (Figure 2B), supporting the notion that corresponding *C1QBP* variants adversely affect the stability of the protein.

**Figure 3:**
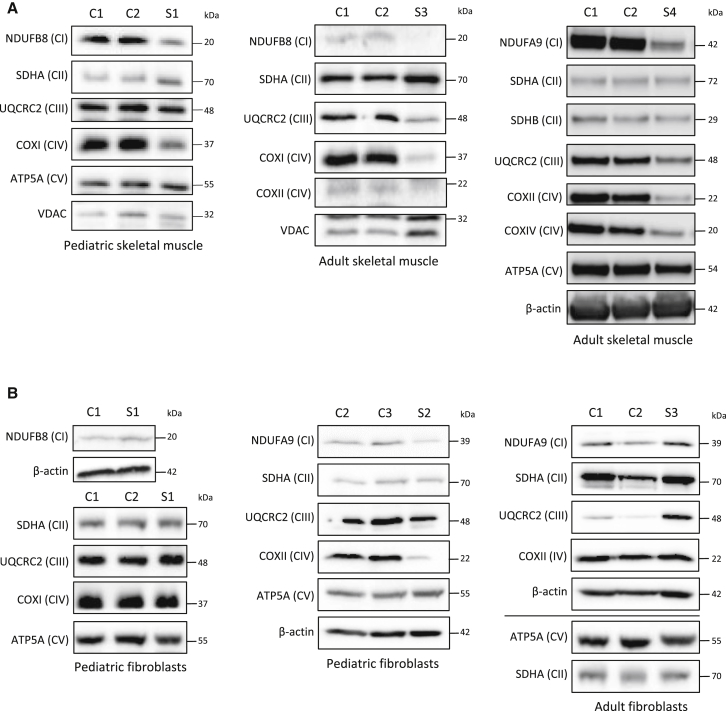
Steady-State Levels of OXPHOS Complex Subunits (A) Western blot analysis of OXPHOS subunits in skeletal-muscle lysates from control individuals (C1 and C2) and probands S1, S3, and S4. (B) Western blot analysis of OXPHOS subunits in skin fibroblasts from probands S1–S3 and age-matched control individuals (C1–C3). OXPHOS-subunit-specific antibodies were used against NDUFB8 or NDUFA9 (CI); SDHA or SDHB (CII); UQCRC2 (CIII); COXI, COXII, or COXIV (CIV); and ATP5A (CV). Cytosolic β-actin and mitochondrial markers porin (VDAC) and SDHA were used as loading controls. All experiments were repeated at least twice, and representative western blots are shown. Number of repeats: (A) n = 3 (S1) and n = 2 (S3 and S4); (B) n = 4 (S1) and n = 2 (S2 and S3).

Consistent with the findings of enzymatic investigations from muscle, a decrease in complex I and complex IV subunits was present in muscle homogenates derived from probands S1, S3, and S4 (Figure 3A). In addition, UQCRC2 was decreased in the muscle of probands S3 and S4 (Figure 3A). In contrast, OXPHOS protein levels were normal or only marginally lowered in fibroblasts from probands S1 and S3, whereas proband S2 showed a marked loss of complex IV subunits (Figure 3B). Surprisingly, the steady-state levels of complex III subunits were higher in the fibroblasts of proband S3 than in control cells (Figure 3B).

**Figure 4:**
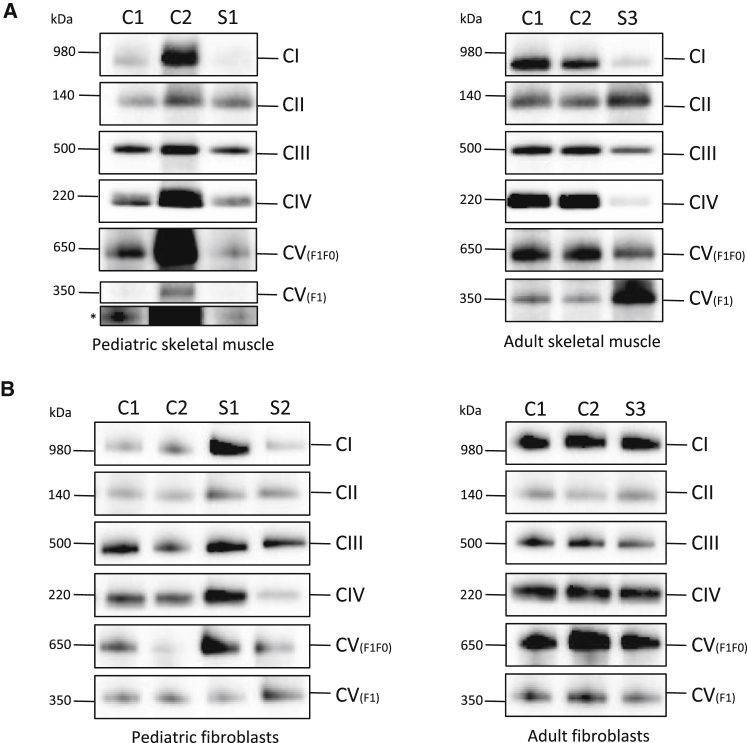
BN-PAGE of OXPHOS Complexes One-dimensional BN-PAGE analysis of OXPHOS complexes was performed on mitochondrial lysates isolated from probands’ (A) skeletal muscle (S1 and S3) and (B) fibroblasts (S1–S3) and age-matched control individuals (C1 and C2). The assembly of OXPHOS complexes was assessed by western blotting with antibodies against NDUFB8 (CI), SDHA (CII), UQCRC2 (CIII), COXI (CIV), and ATP5A (CV). An antibody against complex II subunit SDHA was used as a loading control. The ATP5A antibody detected two bands: the fully assembled complex V (F_0_F_1_) and the soluble F_1_ subunit. The asterisk indicates a longer exposure time for the CV F_1_ subunit. All experiments were repeated at least twice, and representative images are shown. Number of repeats: (A) n = 4 (S1) and n = 2 (S3); (B) n = 4 (S1) and n = 2 (S2 and S3).

Decreased protein levels of subunits of respiratory-chain complexes usually reflect assembly defects, but they can also be suggestive of other mitochondrial disorders, including mitochondrial translation defects. We therefore analyzed the steady-state levels of intact respiratory-chain complexes by BN-PAGE. A decrease in intact steady-state levels of complexes I, IV, and V was present in the muscle of probands S1 and S3 (Figure 4A). Only a slight decrease in complex IV was obvious in proband S1. The level of complex II was normal. In agreement with the decrease in the steady-state levels of complex I and complex IV subunits in the fibroblasts of individual S2, decreased levels of assembled complex I and complex IV were found (Figure 4B). The level of complex III was normal in proband S2 (Figure 4B). No alterations in the steady-state levels of intact complexes were detectable in fibroblasts from probands S1 and S3 (Figure 4B).

### Complementation Studies with Mouse Fibroblasts

**Figure 5:**
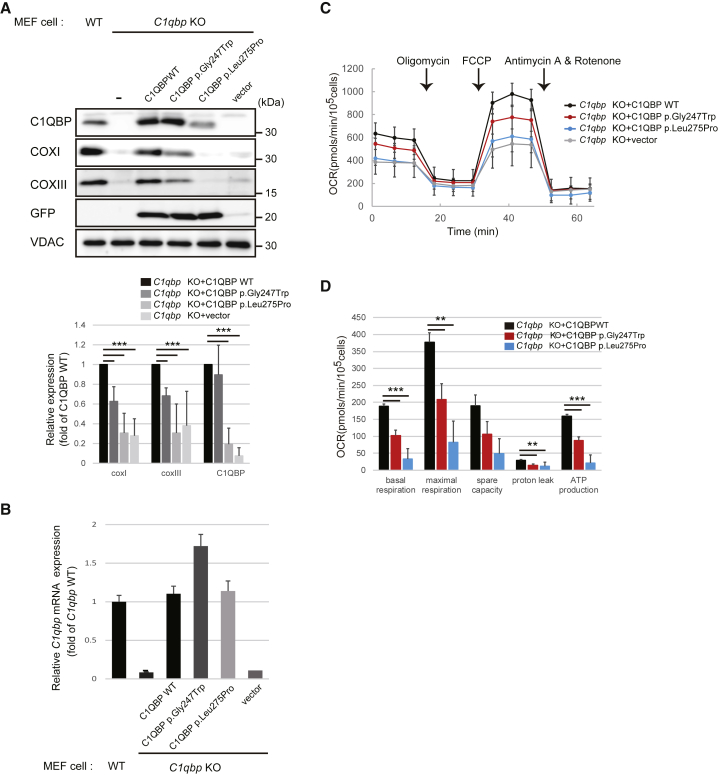
Complementation Studies Using *C1qbp*^*−/−*^ Mouse Fibroblasts (A) Quantification of C1QBP, COXI, and COXIII levels. Top: WT or *C1qbp* KO MEFs were transfected with the pcDNA3-C1qbp-IRES-GFP plasmid for 48 hr. Western blotting on total cell extracts was performed with anti-C1QBP, anti-COXI, anti-COXIII, anti-GFP, and anti-VDAC antibodies. VDAC was used as the loading control. Bottom: the mean ratios of band densities in transfected MEFs from blots are shown. Cells transfected with expression constructs for p.Gly244Trp and p.Leu272Pro variants show significantly lower amounts of mature C1QBP and mitochondrial-DNA-encoded proteins (COXI and COXIII). The results represent the mean ± SD of three independent experiments. ^∗∗∗^p < 0.005 versus WT transfectant. (B) Relative mRNA expression of *C1qbp* alleles normalized to mouse 18S rRNA by quantitative real-time PCR. The results represent the mean ± SD of three independent experiments. (C and D) Oxygen consumption rate (OCR) profile (C) and histogram of *C1qbp* KO cells transfected with WT and mutant *C1qbp* plasmid (D). The histogram shows the basal respiration rate (basal), ATP production rate (ATP), and maximal respiration rate (maximal) calculated from OCR profiles. Data show the mean ± SD of triplicated assays. ^∗∗^p < 0.01 and ^∗∗∗^p < 0.005 versus WT transfectant.

We recently showed that a KO of the orthologous *C1qbp* in MEFs caused impaired mitochondrial respiration associated with reduced levels of respiratory-chain complexes I, III, and IV.^16^ *C1qbp*^*−/−*^ MEFs recapitulate the human disease phenotype by displaying multiple OXPHOS defects. We therefore took advantage of the strong phenotype in the mouse system to investigate the functional properties of the *C1QBP* variants identified in probands S2 and S3. We modeled the variants in the mouse cDNA and expressed them in *C1qbp*^*−/−*^ MEFs. With mitochondrial complex IV subunits I (COXI) and III (COXIII) as markers, only re-expression of mouse WT *C1qbp* rescued the amount of complex IV protein (Figure 5A, upper panel, lane 3), confirming causality between loss of C1QBP and diminished levels of OXPHOS subunits. Expression of *C1qbp* cDNA coding the p.Gly247Trp variant (p.Gly244Trp in mice) resulted in levels comparable to those of the native form but only partially rescued COX subunit levels (Figure 5A, upper panel, lane 4), confirming a functional defect.

Expression of *C1qbp* cDNA encoding the p.Leu275Pro variant (p.Leu272Pro in mice) did not result in a detectable C1QBP and was consequently not able to rescue mitochondrial COXI or COXIII levels (Figure 5A, upper panel, lane 5). GFP expression under the internal ribosome entry site and the *C1qbp* mRNA level were comparable to those of the WT (Figures 5A and 5B), suggesting that the low expression of p.Leu272Pro is due to reduced protein expression or increased protein turnover.

To validate the consequences on respiration, we performed high-resolution respirometry with *C1qbp*-deficient MEFs expressing cDNAs encoding the WT, p.Gly244Trp, p.Leu272Pro, or an empty vector. Reduced basal and maximal respiration, proton leakage, and ATP production were found in cells non-transduced or transduced with the empty vector (Figures 5C and 5D). Consistent with the results of the western blot analysis, p.Gly244Trp resulted in a partial recovery, whereas p.Leu272Pro was in a range similar to that of the empty vector (Figures 5C and 5D).

In summary, complementation with WT, but not mutant, *C1qbp* in *C1qbp*^*−/−*^ MEFs restored C1QBP steady-state level, OXPHOS protein expression, and enzyme activities.

### mtDNA analysis

**Figure 6:**
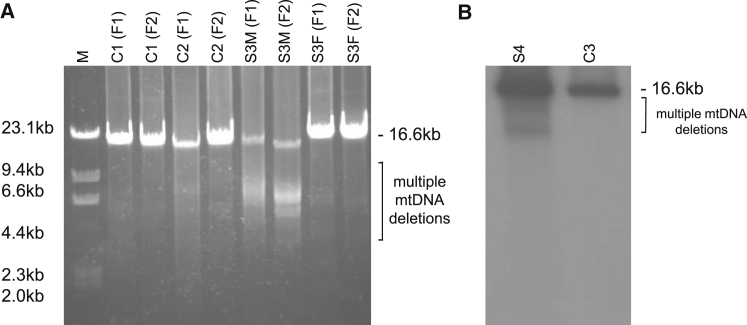
Long-Range PCR and Southern Blot of Individuals with Multiple mtDNA Deletions and Control Individuals (A) Long-range PCR of S3. (B) Southern blot of proband S4. mtDNA was amplified with two primer pairs, giving 16,147 bp (F1) and 15,679 (F2) bp fragments. 5 μL of the amplified mtDNA was loaded onto a 0.7% agarose gel and separated at 80 V for 1 hr. Abbreviations are as follows: S3M, muscle of proband S3; S3F, fibroblasts of proband S3; S4, muscle of proband S4; M, marker λ/HindIII; C1–C3: control cells.

Given that proband S3 presented with PEO, which is often associated with mtDNA rearrangements, analyses of mtDNA copy number and multiple mtDNA deletions were performed in all available muscle and fibroblast cell lines (Table S2). These revealed that mtDNA copy number was 250% higher in the muscle of proband S1 and ∼600% higher in the liver of proband S2 than in control cells, but there was no evidence of mtDNA rearrangements (Table S2). Although the level of mtDNA was within the normal range in muscle samples from probands S3 and S4, long-range PCR and Southern blotting both revealed evidence of multiple mtDNA deletions (Figure 6). Concordant with other investigations of fibroblasts from proband S3, no significant finding (borderline) was obtained in this individual’s cell line.

## Discussion

Here, we report four individuals from unrelated families where biallelic mutations in *C1QBP* cause a combined respiratory-chain enzyme deficiency. All variants are rare with a MAF < 0.00005 in the ExAC Browser, and no other individual in our in-house database of >10,000 WES datasets carries rare biallelic variants in this gene. The finding of four individuals with a defect in mitochondrial energy metabolism (among 2,000 individuals investigated) presents a genome-wide-significant enrichment of biallelic *C1QBP* variants (p < 0.001, Fisher’s exact test) in comparison with samples from non-mitochondrial disorders. In addition, all variants are predicted to be deleterious by several prediction programs.

To validate the impact of the rare *C1QBP* variants at the functional level, we took advantage of three established resources: biopsy tissues, primary fibroblast cell lines from three affected individuals, and *C1qbp*^*−/−*^ MEFs. Mitochondrial localization of C1QBP was shown by immunohistochemical staining in fibroblasts. Immunodecoration using C1QBP-specific antibodies in muscle-biopsy material obtained from all individuals failed to detect C1QBP, indicating a loss of function. However, when investigating the probands’ fibroblasts, we detected low but substantial amounts of C1QBP in all cell lines, most likely reflecting different expression and turnover rates between muscle and fibroblasts. These data argue against a loss of function and for an adverse effect on protein stability of the identified *C1QBP* variants; this could lead to depletion of C1QBP in some tissues with higher turnover rates. In any case, the data provide additional evidence for the functional relevance of the *C1QBP* variants.

All individuals showed a severe combined deficiency of respiratory-chain complexes (I, III, and IV) in muscle or liver homogenates. These findings are in line with observations in yeast and mice models, where the deficiency of the *C1QBP* orthologs resulted in impairment of mitochondrial ATP synthesis or a combined deficiency of respiratory-chain complexes, respectively.^16^, ^17^, ^18^ For both the p.Gly247Trp and p.Leu275Pro variants, this deficiency could be rescued by expression of human WT *C1QBP*. We therefore leveraged the respiratory-chain deficiency in *C1qbp*^*−/−*^ MEFs and employed it to screen for functional complementation by the human mutations. We tested two alleles, and whereas one allele only partially rescued the phenotype of the fibroblasts, the other did not function at all (Figure 5). With these experiments, we provide convincing evidence that the identified *C1QBP* mutations cause a combined respiratory-chain deficiency. Analogous to findings in mice, biallelic loss-of-function *C1QBP* variants might be embryonically lethal in humans.

Mutations in half of the known mitochondriopathy-associated genes result in a combined deficiency of respiratory-chain enzymes.^2^ Combined defects are typically due to deficiencies in mtDNA replication, transcription, or translation. Experiments on KO mouse and KD human cells argued for a role of C1QBP in mitochondrial protein synthesis.^16^, ^19^ Investigations on biopsy tissue from individuals with C1QBP variants confirmed this observation. We found lower levels of various subunits of the respiratory chain in these samples than in age-matched control samples (Figures 3 and 4), whereas RNA expression studies on one cell line did not reveal any alterations in the level of mitochondrial transcripts (data not shown). As expected from earlier complementation studies, a role of C1QBP in protein synthesis seems to be a conserved function of C1QBP in mitochondria.

Interestingly, the yeast KO cells additionally showed a disturbed maintenance of the mitochondrial genome, a feature that has not been observed in higher eukaryotes so far but is also not very specific in yeast given that it is frequently found to be a result of impaired OXPHOS in these cells.^18^ Multiple mtDNA deletions could also explain a combined respiratory-chain deficiency with normal complex II activity. However, multiple deletions were only detectable in the muscle of two probands (S3 and S4), both of whom had a later onset in either childhood or adulthood. Because we found no evidence of variable mtDNA deletions or mtDNA depletion in the early-onset probands S1 and S2, we can be confident that their respiratory-chain deficiency was not caused by a disturbed maintenance of the mitochondrial genome. Nevertheless, in humans there is no evidence that multiple mtDNA deletions appear as a result of impaired OXPHOS, indicating an additional function of C1QBP in the maintenance of the human mitochondrial genome. Given that all cells investigated so far are acute KD or embryonic fibroblasts and, in the case of S1 and S2, are based on biopsies taken in the first week of life, the accumulation of detectable mtDNA deletions in C1QBP deficiency is likely to occur over considerable time. Wang et al. have reported on an interaction between C1QBP and RECQ4, a helicase suggested to participate in mtDNA maintenance.^35^ They describe a RECQ4 mutant that failed to interact with C1QBP, which led to increases in mtDNA copy number and mitochondrial dysfunction. Such scenarios indicate multiple functions of C1QBP in mitochondria. Indeed, a number of mitochondrial interaction partners of C1QBP have been identified by proteomic studies.^36^ They include a number of genes involved in iron-sulfur biogenesis (*BOLA3* [MIM: 614299], *LYRM1* [MIM: 614709], *LYRM2*, *LYRM4* [MIM: 613311], and *LYRM5*) or OXPHOS subunits (*ATP5A1* [MIM: 164360], *C17ORF89*, *COX6B1* [MIM: 124089], *NDUFA4* [MIM: 603833], *NDUFAF4* [MIM: 611776], and *NDUFS3* [MIM: 603846]), as well as factors involved in the maintenance of the mitochondrial morphology (*C2ORF47* [MIM: 617267], *CHCHD2* [MIM: 616244], and *CHCHD10* [MIM: 615903]) and the mitochondrial genome (*TFAM* [MIM: 600438]). The multiple interaction partners of C1QBP point to a multifunctional chaperone role of the protein.^37^

*C1QBP* defects manifested with a spectrum of symptoms ranging from infantile lactic acidosis (in probands S1 and S2) to childhood myopathy, PEO, and later peripheral neuropathy (in proband S3) to adult-onset myopathy with PEO (in proband S4). All individuals presented with major cardiac symptoms, which resulted in early death in the neonatal form and were rather stable in individuals with later presentation. A very recently established cardiomyocyte-specific deletion of *C1qbp* resulted in contractile dysfunction, cardiac dilatation, and cardiac fibrosis and thereby confirmed an important function of C1QBP in the heart.^38^ Decreased COXI and COXIII expression confirmed the mitochondrial dysfunction that resulted in cardiomyopathy at the age of 2 months and a median lifespan of approximately 14 months.^38^

In addition, the two individuals with a late disease onset presented with PEO and variable mtDNA deletions. The clinical manifestation of disorders with deletions in the mitochondrial genome is heterogeneous but often includes PEO.^2^ In the group of disorders with multiple mtDNA deletions, cardiomyopathy is a rare symptom. It is rarely reported in individuals with variants in *POLG* (MIM: 258450)^39^, ^40^, ^41^ and *TWNK* (MIM: 609286)^42^ and has been reported in just a single individual with pathogenic variants in *MGME1* (MIM: 615084).^43^ It is more commonly associated with autosomal-recessive deficiency of *SLC25A4* (cardiomyopathy types of the disease [MIM: 617184 and 615418]), another mtDNA maintenance gene, although variable mtDNA deletions are usually associated with dominant pathogenic variants in this gene.

Numerous functions in various cellular organelles have been reported for C1QBP.^16^ The clinical manifestation of our cohort of probands with C1QBP deficiency was mainly attributed to defects in mitochondrial energy metabolism. No signs of immunologic dysfunction could be associated with the complement system.

Given our observations, the main functions of C1QBP reside within the mitochondrial compartment. However, the exact mechanism leading to a reduction of OXPHOS enzymes remains unclear, especially in the neonatal form.

In summary, we present four individuals with *C1QBP* mutations characterized by combined respiratory-chain deficiency and increased lactate. Disease onset was variable, including intrauterine onset, oligohydramnios, and neonatal cardiomyopathy leading to early death. Later onset in childhood or adulthood was associated with exercise intolerance, PEO, and multiple mtDNA deletions. Cardiomyopathy was found in all forms, which is a relatively unusual presentation in individuals with multiple mtDNA deletions. Peripheral neuropathy seems to be an issue; however, the central nervous system seems to be spared.

## Web Resources

ExAC Browser, http://exac.broadinstitute.org/
GeneReviews, DiMauro, S., and Hirano, M. (1993). Mitochondrial DNA Deletion Syndromes, https://www.ncbi.nlm.nih.gov/books/NBK1203/
MutationTaster, http://www.mutationtaster.org
OMIM, https://www.omim.org
PolyPhen-2, http://genetics.bwh.harvard.edu/pph2/bgi.shtml
PROVEAN, http://provean.jcvi.org/genome_submit_2.php?species=human
RCSB Protein Data Bank, https://www.rcsb.org/pdb/home/home.do
UCSC Genome Browser, https://genome.ucsc.edu/
UnitProt, http://www.uniprot.org/

## Supplemental Data

Document S1. Figure S1 and Tables S1 and S2

Document S2. Article plus Supplemental Data
