# Supplementary material for: Biallelic C1QBP Mutations Cause Severe Neonatal-, Childhood-, or Later-Onset Cardiomyopathy Associated with Combined Respiratory-Chain Deficiencies
Source: Am J Hum Genet. 2017 Sep 21;101(4):525–38. doi: 10.1016/j.ajhg.2017.08.015 (PMC5630164; doi:10.1016/j.ajhg.2017.08.015)
Supplement: Document S1. Figure S1 and Tables S1 and S2 [file mmc1.pdf]

The American Journal of Human Genetics, Volume *101*

Supplemental Data

Biallelic *C1QBP* Mutations Cause Severe Neonatal-, Childhood-, or Later-Onset Cardiomyopathy Associated with Combined Respiratory-Chain Deficiencies

René G. Feichtinger, Monika Oláhová, Yoshihito Kishita, Caterina Garone, Laura S. Kremer, Mikako Yagi, Takeshi Uchiumi, Alexis A. Jourdain, Kyle Thompson, Aaron R. D'Souza, Robert Kopajtich, Charlotte L. Alston, Johannes Koch, Wolfgang Sperl, Elisa Mastantuono, Tim M. Strom, Saskia B. Wortmann, Thomas Meitinger, Germaine Pierre, Patrick F. Chinnery, Zofia M. Chrzanowska-Lightowlers, Robert N. Lightowlers, Salvatore DiMauro, Sarah E. Calvo, Vamsi K. Mootha, Maurizio Moggio, Monica Sciacco, Giacomo P. Comi, Dario Ronchi, Kei Murayama, Akira Ohtake, Pedro Rebelo-Guiomar, Masakazu Kohda, Dongchon Kang, Johannes A. Mayr, Robert W. Taylor, Yasushi Okazaki, Michal Minczuk, and Holger Prokisch

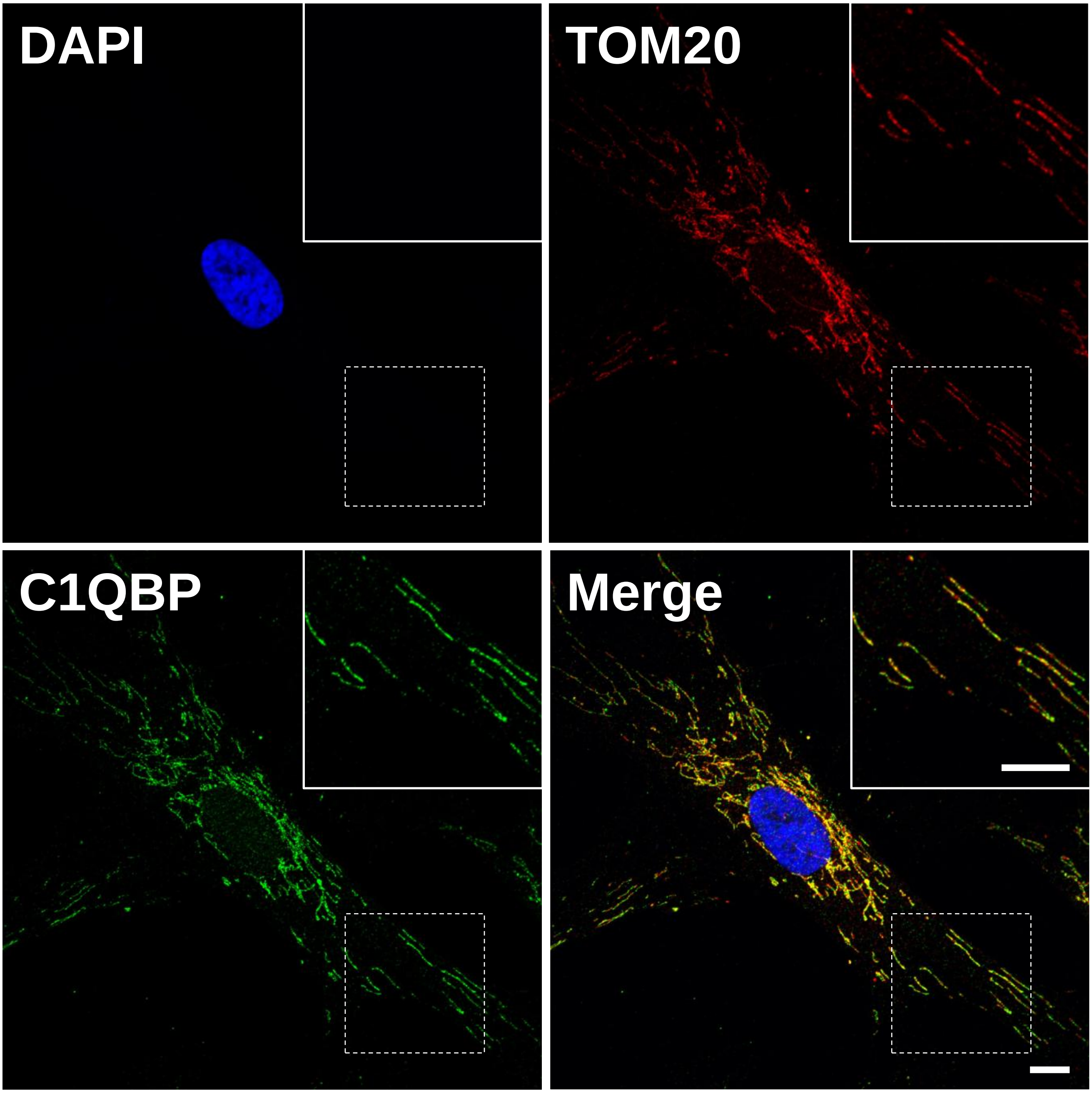

Figure S1. Immunofluorescence analysis of C1QBP localisation in human primary fibroblasts. C1QBP was detected using an antibody to the endogenous protein (green). Mitochondria were detected using anti-TOM20 (red). Nuclei were stained with DAPI (blue). Images were digitally overlaid, with co-localisation of C1QBP with mitochondria appearing in yellow. Insets are magnified views of sections indicated by dashed line. Scale bars: 10 μm.

Table S1. Predicted consequence of *C1QBP* variants

	**P1-Allele 1**	**P1-Allele 2**	**P2-Allele 1**	**P2-Allele 2**	**P3-Allele 1+2**	**P4-Allele 1+2**
Mutation, cDNA (NM_001212.3)	c.557G>C	c.612C>G	c.739G>T	c.824T>C	c.823C>T	c.562_564delTAT
Protein (NP_001203.1)	p.Cys186Ser	p.Phe204Leu	p.Gly247Trp	p.Leu275Pro	p.Leu275Phe	p.Tyr188del
Mutation, genomic (hg19)	chr17:5337008C>G	chr17:5336700G>C	chr17:5336445C>A	chr17:5336360A>G	chr17:5336361G>A	chr17:5337001_5337003delATA
Exon	4	5	6	6	6	4
ExAC frequency	0.000008237 (1/121404)	0.00003296 (4/121360)	0	0	0	0.000008238 (1/121390)
1000 genome frequency	0	0	0	0	0	0
PolypPhen-2 prediction	probably damaging	possibly damaging	probably damaging	probably damaging	probably damaging	NA
PolyPhen-2 score (max. 1)	1	0.922	1	1	0.98	NA
MutationTaster prediction	disease causing	disease causing	disease causing	disease causing	disease causing	disease causing
MutationTaster score	0.999999999012031	0.999996883643345	0.999999999997996	0.99999999999998	0.999999994658642	0.99999999728738
SIFT prediction	Damaging	Damaging	Damaging	Damaging	Damaging	NA
SIFT score (cutoff <0.050)	0.000	0.000	0.000	0.001	0.004	NA
Provean prediction	Deleterious	Deleterious	Deleterious	Deleterious	Deleterious	Deleterious
Provean score (cutoff <-2.50)	-8.67	-5.96	-7.58	-6.29	-3.49	-9.61

PolyPhen-2	http://genetics.bwh.harvard.edu/pph2/bgi.shtml
MutationTaster	http://www.mutationtaster.org
SIFT, Provean	http://provean.jcvi.org/genome_submit_2.php?species=human

Table S2. Biochemical Findings in Individuals with *C1QBP* mutations

S#	Tissue	Histology	Histochemistry	Normalized Mitochondrial Respiratory Chain Activity	Aberrant Activity in %	Molecular Genetics (mtDNA)	
						Multiple mtDNA deletions	mtDNAcopy number
S1	Muscle	NA	RRF<br>COX-deficient fibers (~75% of total biopsy)	I/CS, (**0.028**; n.v.0.104±0.036)<br>II/CS (**0.093**; n.v.0.145±0.047)<br>III/CS (**0.044**; n.v. 0.554±0.345)<br>IV/CS (**0.102**; n.v. 0.124±0.511)	I/CS: 27%<br>II/CS: 64%<br>III/CS: 8%<br>IV/CS: 82%	-	250%
S2	Liver	Lipid droplets	NA	I/CS (**0.0367**; n.r. 0.62-0.71)<br>II/CS (**0.96**; n.r. 2.69-2.74)<br>III/CS (**0.069**; n.r. 0.31-0.63)<br>IV/CS (**0.0024**; n.r.0.019-0.028)<br>CS/mg protein (38.6, n.r. 27.9-49.8)	I/CS: 6%<br>II/CS: 36%<br>III/CS: 22%<br>IV/CS: 13%<br>CS/mg protein: 138%	-	599%
	Fibroblasts	NA	NA	I/CS (0.323; n.r. 0.267-0.792)<br>II/CS (0.311; n.r. 0.287-0.875)<br>III/CS (0.066; n.r. 0.058-0.28)<br>IV/CS (**0.011**; n.r. 0.014-0.064)<br>CS/mg protein (**312.6**; n.r. 82.4-139)	I/CS: nrl<br>II/CS: nrl<br>III/CS: nrl<br>IV/CS: 79%<br>CS/mg protein: 379%	-	NA
S3	Muscle	NA	RRF<br>COX-deficient fibers	I/CS (**0.02**; n.r.0.17-0.31)<br>I+III/CS (**0.15**; n.r.0.24-0.81)<br>III/CS (**0.17**; n.r. 2.12-3.09)<br>IV/CS (**0.15**; n.r. 2.59-3.12)<br>CS/mg protein (**511**; n.r. 150-325)	I/CS: 12%<br>I+III/CS: 63%<br>III/CS: 8%<br>IV/CS: 6%<br>CS/mg protein: 341%	+ (long range)	nrl
	Fibroblasts	NA	NA	I/CS (0,08; 0,06; n.r. 0,05-0,09)<br>I+III/CS (**0,2**; 0,48; n.r. 0,24-0,58)<br>II/CS (0,43; 0,48; n.r. 0,37-0,48)<br>II+III/CS (0,67; 0,66; n.r. 0,39-0,72)<br>III/CS (1,21; 1,23; n.r. 1,17-1,99)<br>IV/CS (**1,22**; **1,25**; n.r. 1,30-1,68)<br>V/CS (0,24; **0,42**; n.r. 0,15-0,39)<br>CS/mg protein (**207**; 442; n.r. 242-590)	I/CS: nrl<br>I+III/CS: nrl<br>II/CS: nrl<br>II+III/CS: nrl<br>III/CS: nrl<br>IV/CS: 95%<br>V/CS: nrl<br>CS/mg protein: nrl	NA	NA
S4	Muscle	Fiber size variability, central nuclei, increased connective tissue	Isolated COX-deficient fibers	I/CS (**0.06**; n.v. 0.11±0.03)<br>I+III/CS (**0.14**; n.v. 0.27±0.05)<br>II/CS (**0.04**; n.v. 0.07±0.01)<br>II+III/CS (**0.03**; 0.09±0.02)<br>IV/CS (**0.16**; 0.35±0.04)<br>CS/mg protein (**151.7**; n.v. 137.3±15)	I/CS: 55%<br>I+III/CS: 52%<br>II/CS: 57%<br>II+III/CS: 33%<br>IV/CS: 46%<br>CS/mg protein: 111%	+ (Southern Blot)	nrl

S#=subject number; NA= not available, RRF= ragged red fibers; I= NADH dehydrogenase; II= succinate dehydrogenase; III= CoQH2-cytochrome c reductase; IV= cytochrome c oxidase; CS= citrate synthase; mg= milligram; nrl= normal; -=absent; += present; COX= cytochrome c oxidase; n.r. = normal range; n.v. = normal values (mean ± standard deviation (SD)). The percent were either calculated as percent of the lower range value or percent of the mean. Reported values are marked in bold. Depending on the method and sample preparation used, the range of control values can vary by an order of magnitude between different metabolic laboratories. (Gellerich, F.N., Mayr, J.A., Reuter, S., Sperl, W., and Zierz, S. (2004). The problem of interlab variation in methods for mitochondrial disease diagnosis: enzymatic measurement of respiratory chain complexes. Mitochondrion 4, 427-439.)
